# Up-regulation of collagen type V alpha 2 (*COL5A2*) promotes malignant phenotypes in gastric cancer cell via inducing epithelial–mesenchymal transition (EMT)

**DOI:** 10.1515/med-2022-0593

**Published:** 2023-01-16

**Authors:** Yanfeng Jin, Xinyan Song, Xuankai Sun, Yan Ding

**Affiliations:** Department of Gastroenterology, Yantai Yuhuangding Hospital, Yantai, China; Pharmacy of Laishan Branch, Yantai Yuhuangding Hospital, Yantai, China; Department of Radiation, Yantai Yuhuangding Hospital, Yantai, China; Department of Surgical Intensive Care Unit, Yantaishan Hospital, No. 10087 Keji Avenue, Laishan District, Yantai, Shandong 264003, China

**Keywords:** collagen, gastric cancer, oncogenic, mesenchymal markers

## Abstract

Recent studies have reported that collagen type V alpha 2 (*COL5A2*) is a hub gene and associated with the prognosis of gastric cancer (GC) patients, playing an important role in GC. In this study, we aim to fathom out the biological roles of *COL5A2* and its relevant mechanism in GC. Oncomine, gene expression profiling interactive analysis, and UALCAN were used to explore the effects of *COL5A2* on GC. Cell counting kit-8 assay, colony formation assay, and transwell assay were conducted to investigate the biological behaviors of GC cell lines AGS and SGC-7901. Quantitative reverse transcription polymerase chain reaction and western blot were performed to determine gene and protein expressions. *COL5A2* expression was up-regulated and negatively correlated with survival percentage of GC patients. *COL5A2* expression was notably elevated in high stage and high grade of GC. Down-regulation of *COL5A2* inhibited proliferation, migration, and invasion of AGS and SGC-7901 cells. *COL5A2* induced epithelial–mesenchymal transition (EMT) by promoting the expressions of mesenchymal markers (SNAI1, SNAI2, TWIST, VIM, and MMP2), thereby facilitating the malignant phenotypes of GC. *COL5A2* plays an oncogenic role in GC and has potential to predict the progression and prognosis of GC patients.

## 1 Introduction

As a malignant tumor, gastric cancer (GC) is reported to be the second leading cause of cancer-related deaths globally, and there are 1,089,103 new cases (5.6% of total cases) and 768,793 deaths (7.7% of total cancer deaths) in 2020 globally [1]. China is the hardest-hit country, with 42.6% of the global incidence and 45% of all GC-related mortality [2]. Due to the lack of effective and safe screening methods and obvious signs at an early stage, patients with GC are already at an advanced stage when first diagnosed, and they face with high risks of metastasis and recurrence after treatment [2]. Although great efforts have been made in the treatment of GC, such as surgery and adjuvant chemotherapy, the prognosis of GC patients has been slightly improved [3,4]. The 5-year survival rate of patients at advanced stage is extremely low, only 20% with the median survival of less than half a year [4]. Therefore, much attention should be paid to the early diagnosis of GC. Recently, scientists have devoted to applying molecular markers in the improvement of cancer diagnosis and therapeutic strategies [5]. However, limited progress has been achieved in early diagnosis and effective therapy for GC. While many biomarkers for GC have been reported, such as carbohydrate antigen (CA) 72-4, alpha-fetoprotein, and CA12-5, carcinoembryonic antigen and CA19-9 are still the most frequently used biomarkers for GC in clinical practice [6,7]. Almost all of the patients with advanced GC cannot be treated with a targeted therapy. Currently, no diagnostic markers are available for secondary prevention. Thus, it is of great significance to dig out the GC-associated molecules and the underlying mechanisms for the management of this disease.

Collagen proteins are the major component of extracellular matrix (ECM) with the highest protein level in mammalian cells [8]. In vertebrates, at least 28 types of collagen are identified, which play various roles in scaffold, fibrosis, and adhesion of tissues [9]. Many studies have revealed that up-regulation and down-regulation of collagens are both involved in the progression of cancers by regulating the remodeling of ECM [10,11]. Collagen type V (COL5), a type of fibrillar collagens, is mainly expressed in bone, dermis, cornea, and placenta co-distributed with type I collagens [12]. COL5 includes three major subunits, including COL5A1, COL5A2, and COL5A3, which can bind to three different polypeptide α chains [12]. COL5 collagens play essential roles in tissue scaffold and cell adhesion via forming heterotrimer or homotrimer [13]. Recently, as one of the best-studied collagens, COL5A2 has been reported to be aberrantly expressed in various cancers, such as breast cancer [14], ovarian cancer [15], adenomas [16], and bladder cancer [17]. Of note, multiple bioinformatics analyses have revealed that *COL5A2* is a hub gene involved in the prognosis of GC patients, with a vital role in GC [4,18–21]. In addition, *COL5A2* level may be a risk factor for GC, and *COL5A2* may act as a potential clinical biomarker for GC and renal metastasis [22]. However, there are no experimental data supporting this notion, and the relevant mechanism remains ambiguous.

Therefore, in this study, we aimed to explore biological roles of *COL5A2* and the potential molecular mechanism in GC. We found that *COL5A2* was highly expressed in GC tissues and cells. Additionally, the expression of *COL5A2* was associated with grades and stages of GC as well as the survival percentage of patients. Loss-of-function assay revealed that *COL5A2* played a promotive role in GC cell proliferation and mobility *in vitro*. Besides, we clarified the relevant mechanism via which *COL5A2* could facilitate the protein expressions of pro-epithelial–mesenchymal transition (EMT)-related genes, including *SNAI1*, *SNAI2*, *TWIST*, *VIM*, and *MMP2*.

## 2 Materials and methods

### 2.1 Oncomine database analysis

The expression of *COL5A2* mRNA was analyzed in Oncomine database (https://www.oncomine.org), a cancer microarray online database. Four reporters, including 2591643, 221730_at, ILMN_1729117, and IMAGE:429203, were used for the analysis of *COL5A2* expression in GC.

### 2.2 Gene expression profiling interactive analysis (GEPIA) database

The mRNA expression of *COL5A2* was also analyzed by a web-based tool GEPIA in 408 GC tissues and 211 normal tissues from the Cancer Genome Atlas (TCGA) database and in 408 GC tissues and 36 normal tissues from GTEx database. The correlation between *COL5A2* expression and survival of GC patients was assessed by Kaplan–Meier (KM) curves plotted by GEPIA. Stage plot was generated to analyze *COL5A2* expression in four stages of GC. The correlation between *COL5A2* and metastasis-related genes (*SNAI1*, *SNAI2*, *TWIST*, *VIM*, and *MMP2*) was evaluated using GEPIA.

### 2.3 UALCAN database

The mRNA expression of *COL5A2* based on sample types, individual cancer stages, and tumor grades were analyzed by UALCAN (http://ualcan.path.uab.edu), an interactive web resource.

### 2.4 Cell culture

GC cell lines including AGS and SGC-7901 and normal cell line GSE-1 were purchased from Chinese Academy of Medical Sciences cell bank (Shanghai, China). All the cells were cultured in Dulbecco’s Modified Eagle’s Medium (DMEM), which was supplemented with 10% fetal bovine serum (FBS) and penicillin–streptomycin solution. Cells in logarithmic phase were inoculated into a six-well plate for further studies.

### 2.5 Transfection assay

*COL5A2* expression was down-regulated by transfecting small interfering RNA (siRNA) targeting COL5A2 (si-*COL5A2*: si-*COL5A2*#1, 5′-CCATCCAGTGTACCACGTAAA-3′; si-*COL5A2*#2, 5′-CCAGGCTCCATAGGAATCAAA-3′) into cells with the help of Lipofectamine2000 (Thermo Scientific, USA) according to the manufacture’s guidance. The si-con (5′-ACGAGACACGAACGGAGAATT-3′) was used as the control. The two *COL5A2* siRNAs and si-con were obtained from GenePharma (Shanghai, China). The overexpression plasmid of *COL5A2* and empty vector purchased from GenePharma (Shanghai, China) were used to transfect AGS and SGC-7901 cells using Lipofectamine2000 (Thermo Scientific, USA) in strict accordance with the manufacturer’s guidance.

### 2.6 Quantitative reverse transcription polymerase chain reaction (qRT-PCR)

The total RNA was isolated using TRIzol reagent (Thermo Scientific, USA) under the manufacturer’s instructions. Complementary DNAs (cDNAs) were reversely transcribed from extracted RNAs with RevertAid First Strand cDNA Synthesis Kit (Thermo Scientific, USA), followed by the qRT-PCR assay with TB Green Premix Ex Taqt II (TaKaRa, Japan). The 2^–ΔΔCt^ method was applied to determine the level of mRNA, with normalization to glyceraldehyde-3-phosphate dehydrogenase (GAPDH) mRNA expression. The primers used were listed as follows: *COL5A2* (Forward: 5′-CAGGCTCCATAGGAATCAGAGG-3′, Reverse: 5′-CCAGCATTTCCTGCTTCTCCAG-3′) and *GAPDH* (Forward: 5′-GTCTCCTCTGACTTCAACAGCG-3′, Reverse: 5′-ACCACCCTGTTGCTGTAGCCAA-3′).

### 2.7 Western blot

Total proteins isolated from the cells were quantified by bicinchoninic acid protein assay kit (Beyotime, China), followed by the separation with sodium dodecyl sulfate-polyacrylamide gel electrophoresis. Then the proteins were electrically transferred onto a polyvinylidene fluoride membrane, blocked with 5% skim milk, incubated with primary antibodies, washed with tris-buffered saline with tween-20 and maintained with appropriate secondary antibodies. Then BeyoECL Plus substrate (Beyotime, China) exploited to visualize the target protein band. The levels of proteins were analyzed on an ImageQuant LAS 4000 system (GE Healthcare) and normalized to GAPDH expression. The primary antibodies included those against COL5A2 (dilution ratio: 1:1,000, #PA5-14245; Invitrogen, Thermo Scientific, USA), SNAI1 (dilution ratio: 1:1,000, ab216347; Abcam, UK), SNAI2 (dilution ratio: 1:1,000, ab51772; Abcam, UK), TWIST (dilution ratio: 1:1,000, ab50887; Abcam, UK), VIM (dilution ratio: 1:1,000, ab92547, Abcam, UK), MMP2 (dilution ratio: 1:1,000, ab92536; Abcam, UK), and GAPDH (dilution ratio: 1:1,000, ab8245; Abcam, UK).

### 2.8 Cell counting kit-8 (CCK-8) assay and colony formation assay

Cell proliferation was detected by CCK-8 kit (Beyotime, China) and colony formation assay. For CCK-8 assay, we seeded the transfected cells in a six-well plate at a density of 1,000 cells/well. CCK-8 reagent was added for surveillance of cell viability at 0, 24, 48, and 72 h. After culture for another 1.5 h, the optical density (OD) of cells was determined at a wavelength of 450 nm with a microplate reader.

For colony formation assay, the transfected cells were first seeded in a 60 mm dish (400 cells/dish) and cultured at 37℃ for 1–2 weeks. When macroscopic colonies appeared, cell culture was terminated. After the cells were fixed and stained, the number of the colonies was counted under an optical microscope.

### 2.9 Transwell assay

After 48 h transfection, the cells were suspended in serum-free DMEM (1 × 10^4^ cells/100 μL) and inoculated into the upper layer of transwell chamber. The lower layer was filled with medium containing 10% FBS. After maintenance at 37℃ overnight, the migrated cells were fixed and stained. The number of cells in five randomly selected microscopic vision fields was then counted and the cells were imaged under an Olympus BX41 light microscope (Olympus Corporation) at a magnification of ×200. For invasion assay, the upper layer of transwell chamber was coated with Matrigel.

### 2.10 Statistical analysis

SPSS 22.0 (IBM, USA) and GraphPad Prism7 (GraphPad, USA) were used to perform statistical analysis. Student’s *t*-test and one-way analysis of variance were utilized to compare the difference between the two groups and among the three or more groups, respectively. When *P*-value was less than 0.05, the difference was statistically significant.

## 3 Results

### 3.1 *COL5A2* was highly expressed in GC patients

**Figure 1:**
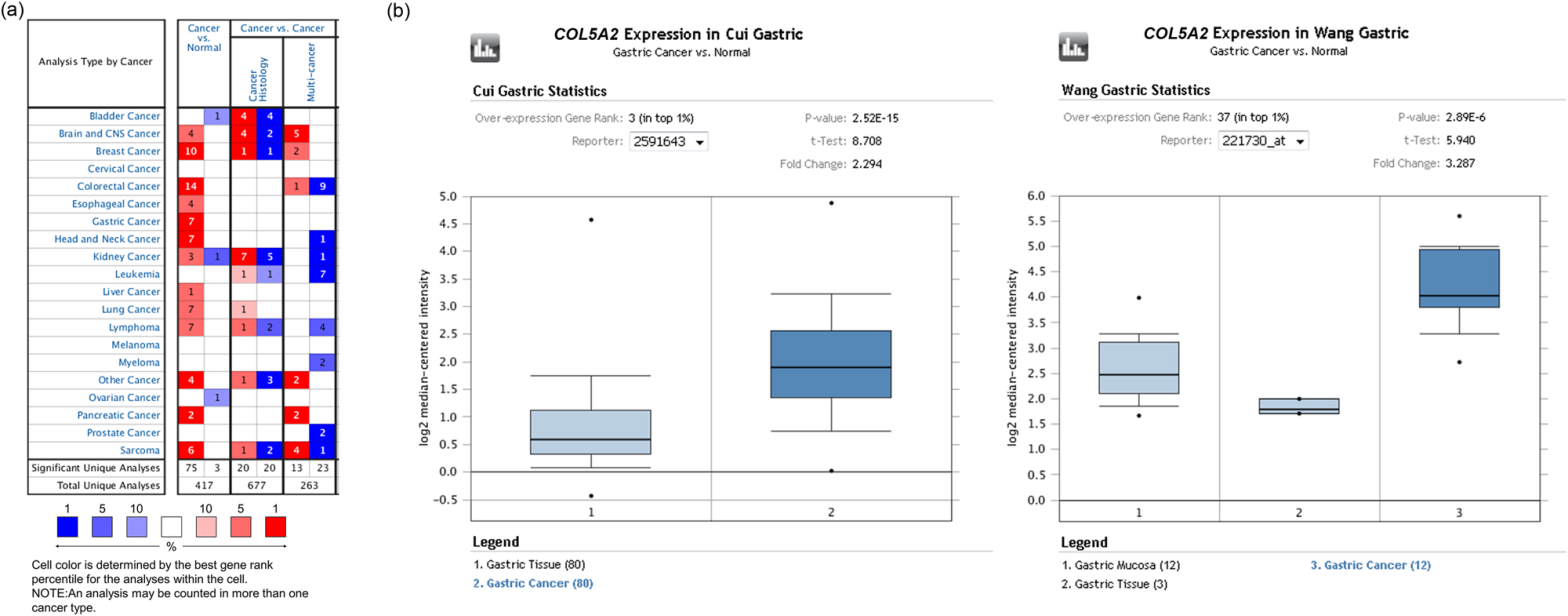
Oncomine analysis revealed that the mRNA expression of *COL5A2* was up-regulated in GC. (a) mRNA expression of *COL5A2* in a variety of cancers was analyzed, and *COL5A2* expression was up-regulated in most tumor types. (b) mRNA expression of *COL5A2* was evidently elevated in GC tissues.

**Figure A1:**
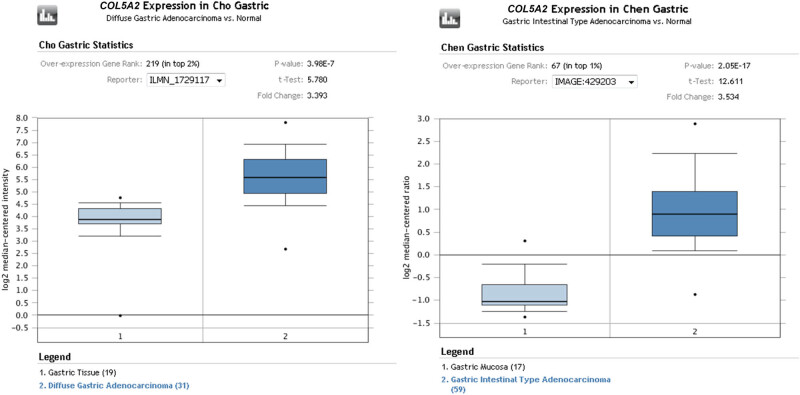
Oncomine analysis revealed that the mRNA expression of *COL5A2* was up-regulated in GC.

**Figure A2:**
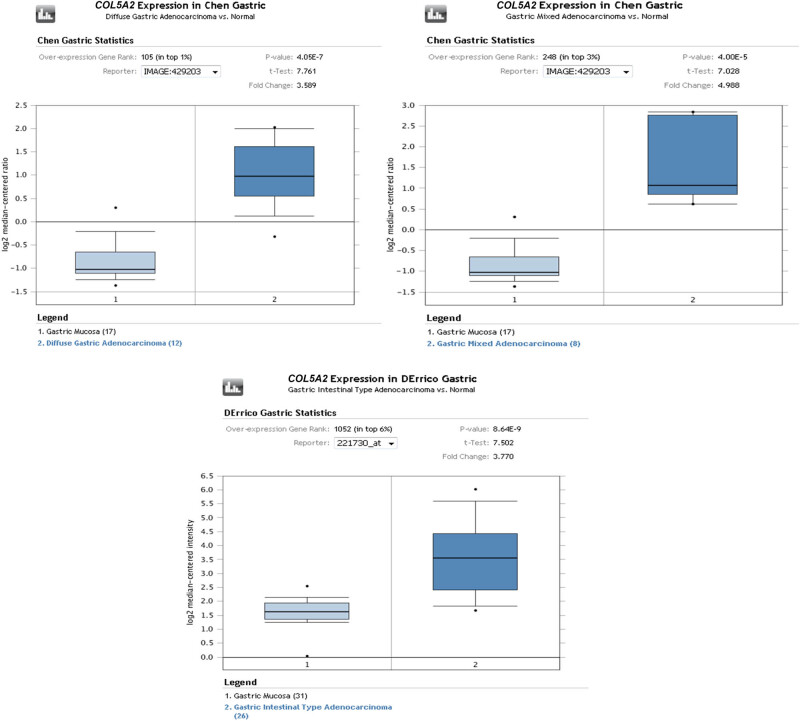
The mRNA expression of *COL5A2* was evidently elevated in three common histological subtypes of gastric cancer including intestinal, diffused and mixed gastric adenocarcinomas in Chen and DErrico datasets.

To figure out the role of *COL5A2*, we first analyzed the expression of *COL5A2* using Oncomine database, GEPIA, and UALCAN. The results indicated that highly-expressed *COL5A2* appeared in 18 of 20 tumor types, especially in breast, gastric, colorectal, and head and neck cancers (Figure 1a), which was consistent with the findings of several previous studies [18,19]. The mRNA expression of *COL5A2* showed 2.294-fold (*P* = 2.52 × 10^−15^) increase in GC samples (*n* = 80) as compared with that in normal tissues (*n* = 80, Figure 1b). Besides, *COL5A2* expression was elevated in three common histological subtypes of GC, including intestinal, diffused, and mixed gastric adenocarcinomas, as compared with that in gastric tissues or mucosa in Wang, Cho, Chen, and DErrico datasets (Figure 1b, Figures A1 and A2, *P* < 0.05).

**Figure 2:**
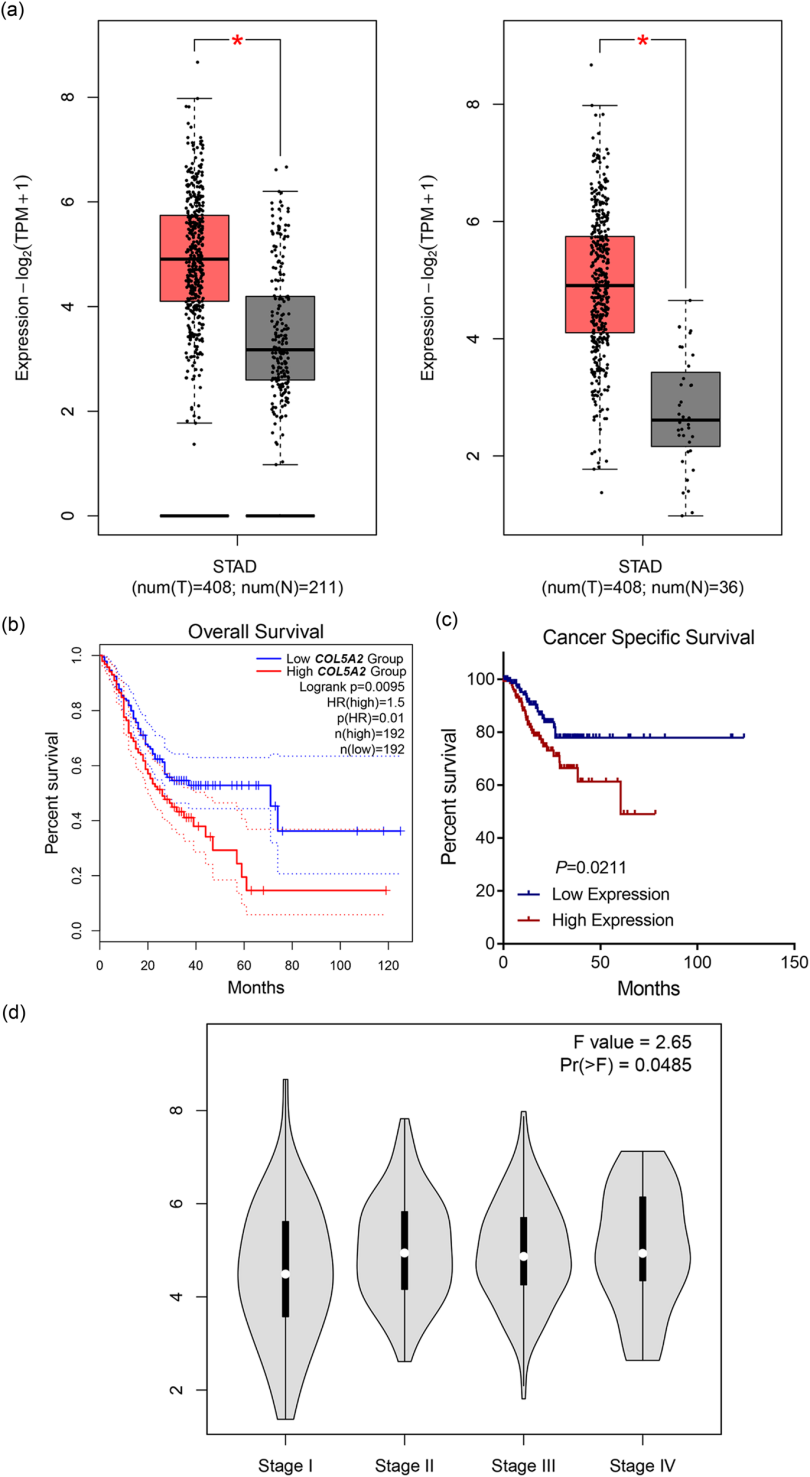
GEPIA analysis revealed that the mRNA expression of *COL5A2* was up-regulated in GC. (a) mRNA expression of *COL5A2* was analyzed in GEPIA combined with TCGA (left) or GTEx database (right); *P < 0.05. (b) KM survival curves of the overall survival for *COL5A2* were plotted, and patients in *COL5A2* high expression group had a shorter survival than those in *COL5A2* low expression group. (c) KM survival curves of the GC-specific survival for *COL5A2* was plotted, and patients in *COL5A2* high expression group had a shorter survival than those in *COL5A2* low expression group. (d) Association between *COL5A2* expression and tumor stages of GC was analyzed by stage plot.

Next, the mRNA level of *COL5A2* was also analyzed by GEPIA with TCGA and GTEx databases. As depicted in Figure 2a, the expression of *COL5A2* was determined to be higher in GC tissues than that in normal tissues (*P* < 0.05). Then, the survival information of 384 GC patients was classified into two groups according to the median expression level of *COL5A2*. The association between *COL5A2* expression and overall survival of GC patients was evaluated by KM curves. Figure 2b revealed that the overall survival time was shorter in *COL5A2* high expression group compared with that in C*OL5A2* low expression group (*P* < 0.01), and the cancer-specific survival time was shorter in *COL5A2* high expression group as compared to that in *COL5A2* low expression group (Figure 2c, *P* < 0.05). Besides, the mRNA expression of *COL5A2* in four stages of GC was analyzed as well. The stage plot displayed that patients in stages II, III, and IV expressed higher level of *COL5A2* than those in stage I (Figure 2d, *P* < 0.05).

**Figure 3:**
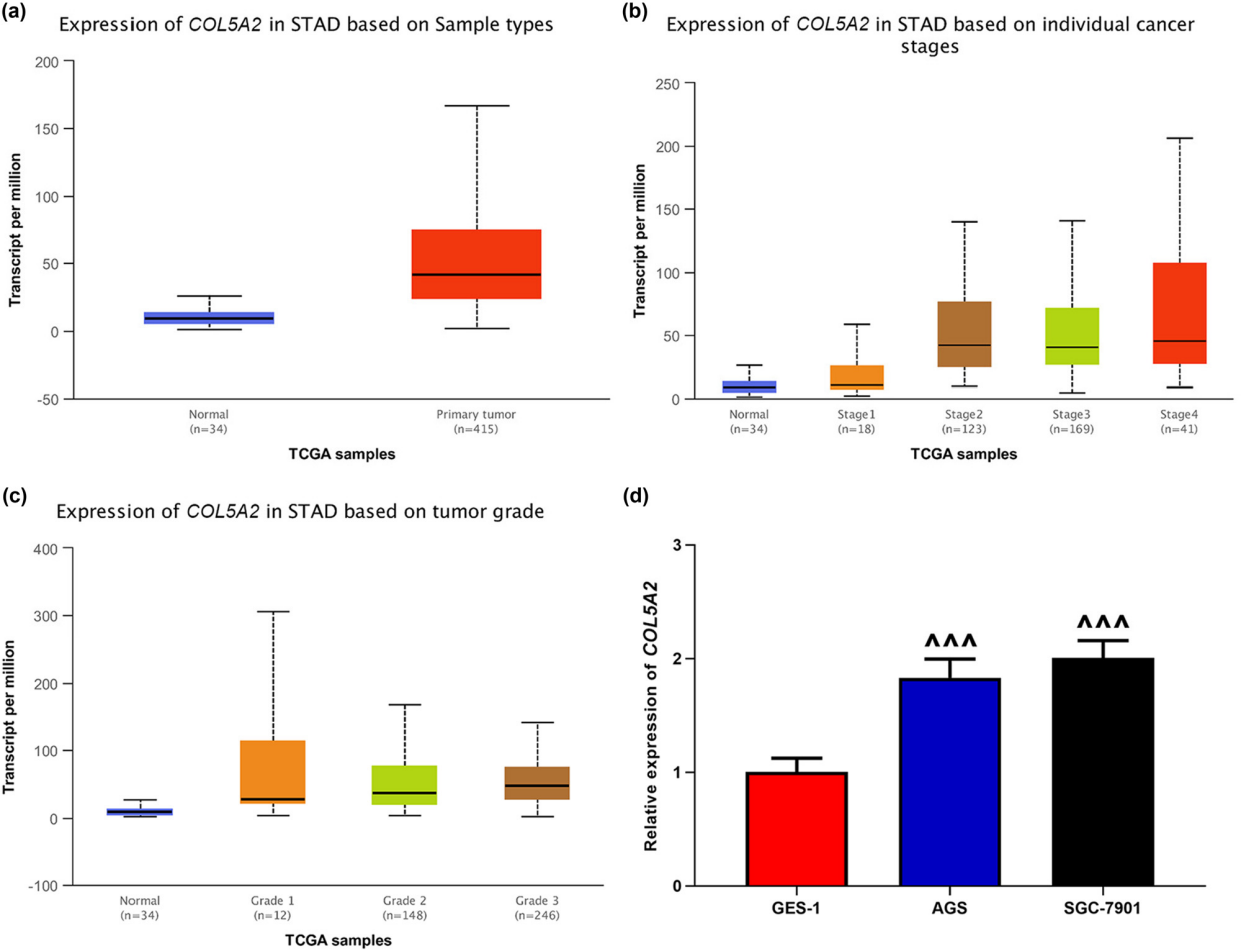
UALCAN analysis revealed that the mRNA expression of *COL5A2* was up-regulated in GC. (a–c) Expression of *COL5A2* was analyzed based on sample types (a), cancer stages (b), and tumor grades (c); *P* < 0.05. Stomach adenocarcinoma (STAD). (d) mRNA expression of *COL5A2* was detected in AGS, SGC-7901, and GES-1 cells. Data from three independent experiments were exhibited as mean ± standard deviation (SD). ^∧∧∧^ *P* < 0.001 vs GES-1.

Further, we analyzed *COL5A2* expression in GC using UALCAN based on sample types, cancer stages, and grades with TCGA database. The results further confirmed that *COL5A2* expression was markedly up-regulated in 415 GC tissues relative to that in 34 normal tissues (Figure 3a, *P* < 0.05). When classified by cancer stages, *COL5A2* expression level was notably higher in stages II, III, and IV subgroups than that in stage I and normal subgroups (Figure 3b, *P* < 0.05). Based on tumor grade, *COL5A2* expression varied in grades I, II, and III subgroup patients (Figure 3c). Collectively, our results demonstrated that *COL5A2* expression was up-regulated and associated with the prognosis and stages of GC patients, signifying the pivotal roles of *COL5A2* in GC.

### 3.2 *COL5A2* expression was up-regulated in GC cells

**Figure 4:**
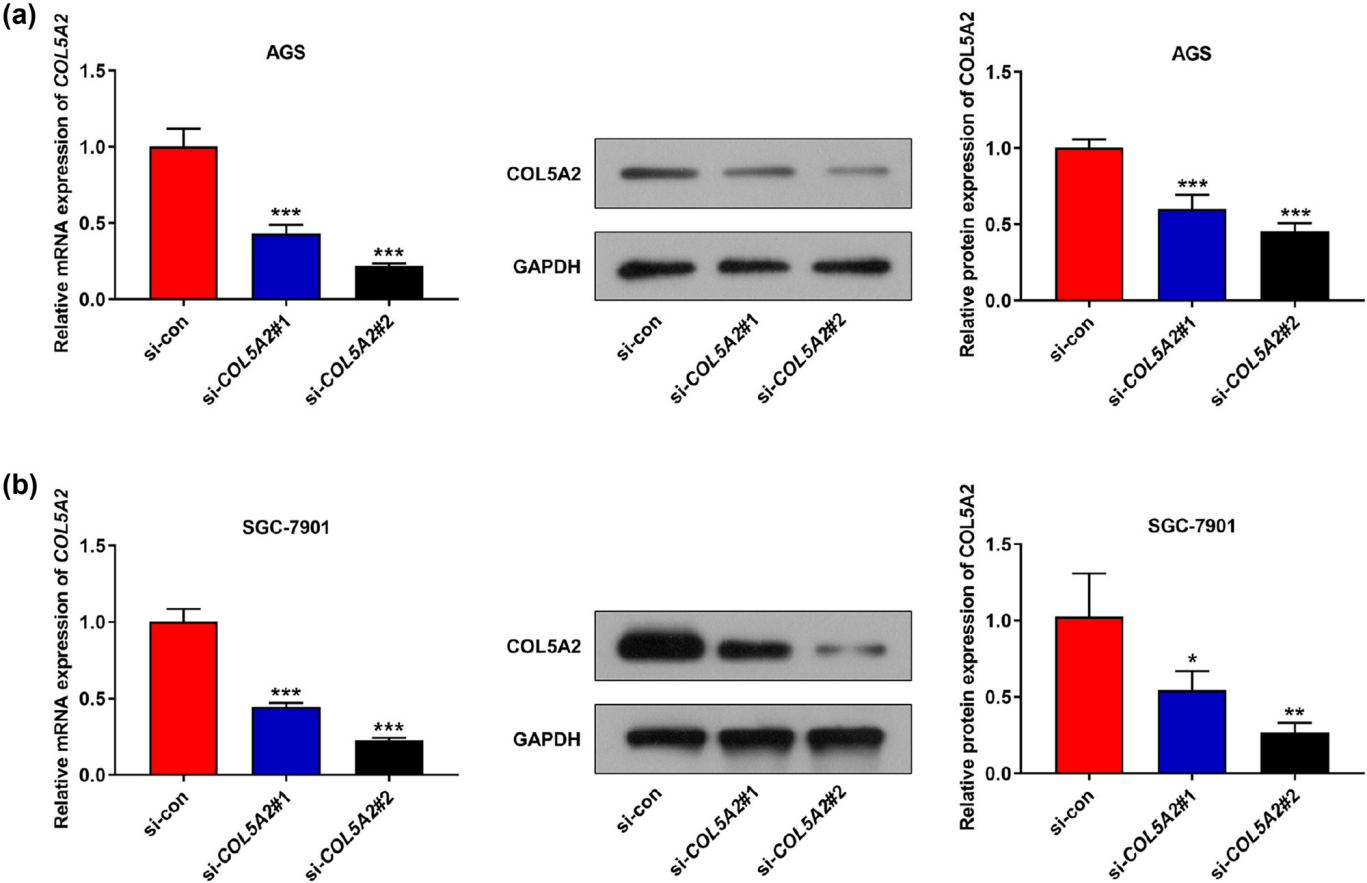
*COL5A2* expression was down-regulated in GC cells after transfected with si-*COL5A2*. (a) mRNA and protein expressions of COL5A2 were analyzed in si-*COL5A2* (si-*COL5A2*#1 or si-*COL5A2*#2)- or si-con-transfected AGS cells. (b) mRNA and protein expressions of COL5A2 were observed in si-*COL5A2* (si-*COL5A2*#1 or si-*COL5A2*#2) or si-con-transfected SGC-7901 cells. Data from three independent experiments were expressed as mean ± SD. *P < 0.05, **P < 0.01 and ***P <0.001 vs si-con group.

In order to investigate the roles of *COL5A2* in GC *in vitro*, two GC cell lines, including AGS and SGC-7901, were singled out. qRT-PCR analyses revealed that the expression of *COL5A2* in AGS and SGC-7901 cells was higher than that in the normal cell line GES-1 (Figure 3d, *P* < 0.01), which was consistent with the result of bioinformatics analysis. Next, we performed loss-of-function assay by knocking down *COL5A2* level with si-*COL5A2*#1 and si-*COL5A2*#2 in GC cell lines. Figure 4a mirrored that the mRNA and protein expressions of COL5A2 were prominently decreased in AGS cells transfected with si-*COL5A2*#1 and si-*COL5A2*#2 as compared with those in cells transfected with si-con (*P* < 0.01). Moreover, the same results were obtained in SGC-7901 cells (Figure 4b, *P* < 0.01). Meanwhile, si-*COL5A2*#2 displayed better efficiency in AGS and SGC-7901 cells than in si-*COL5A2*#1 (Figure 4a and b, *P* < 0.01). Therefore, we selected si-*COL5A2*#2 to transfect GC cells for further analyses.

### 3.3 Knockdown of *COL5A2* inhibited the proliferation, migration, and invasion of GC cells

**Figure 5:**
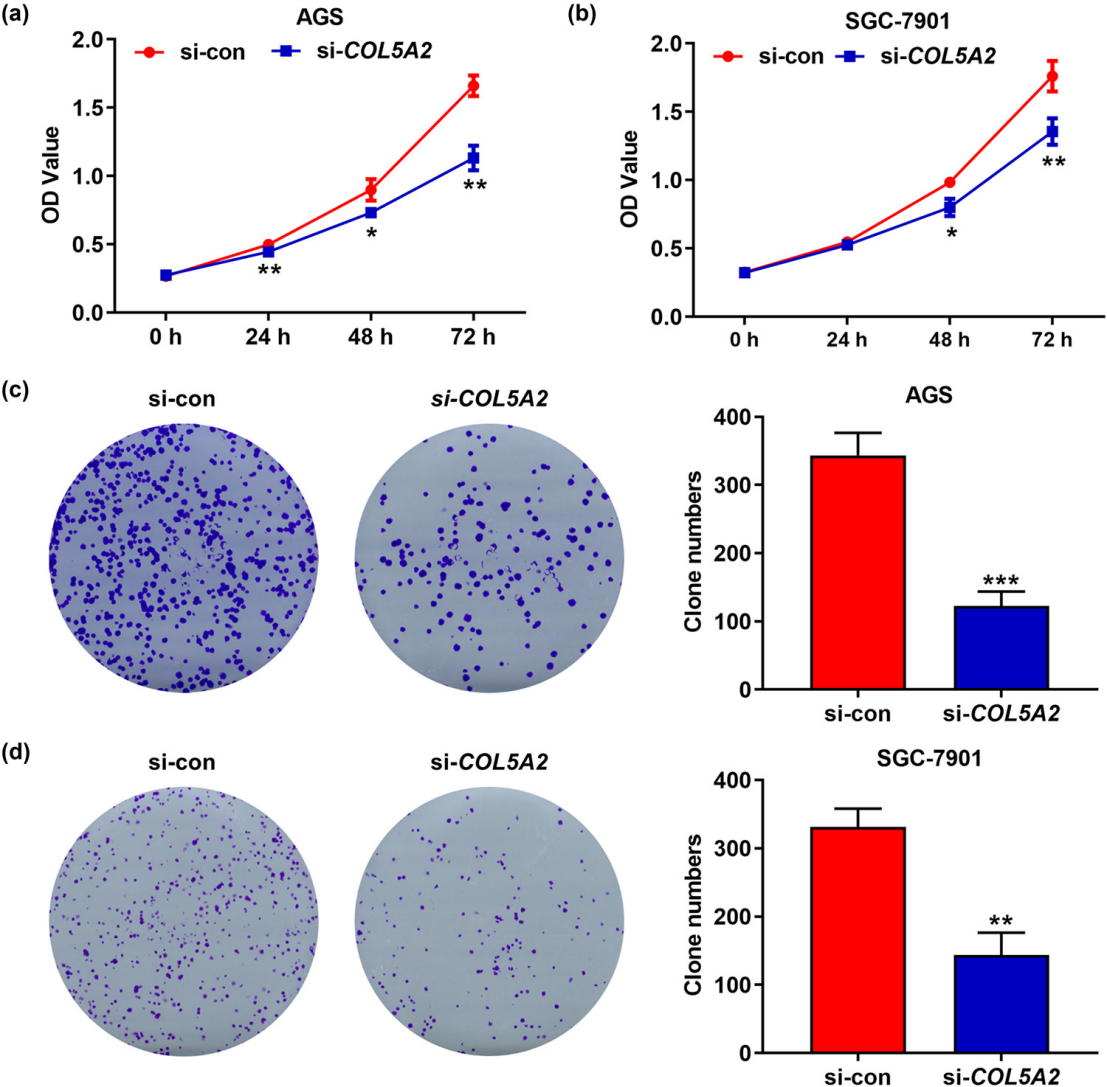
Knockdown of *COL5A2* inhibited proliferation of GC cells. (a and b) OD values in si-*COL5A2*- or si-con-transfected GC cell lines including AGS (a) and SGC-7901 (b) were determined by CCK-8 assay. (c and d) Colony numbers of si-*COL5A2*- or si-con-transfected GC cell lines including AGS (c) and SGC-7901 (d) were analyzed by colony formation assay. *P < 0.05, **P < 0.01 and ***P < 0.001 vs si-con group.

**Figure 6:**
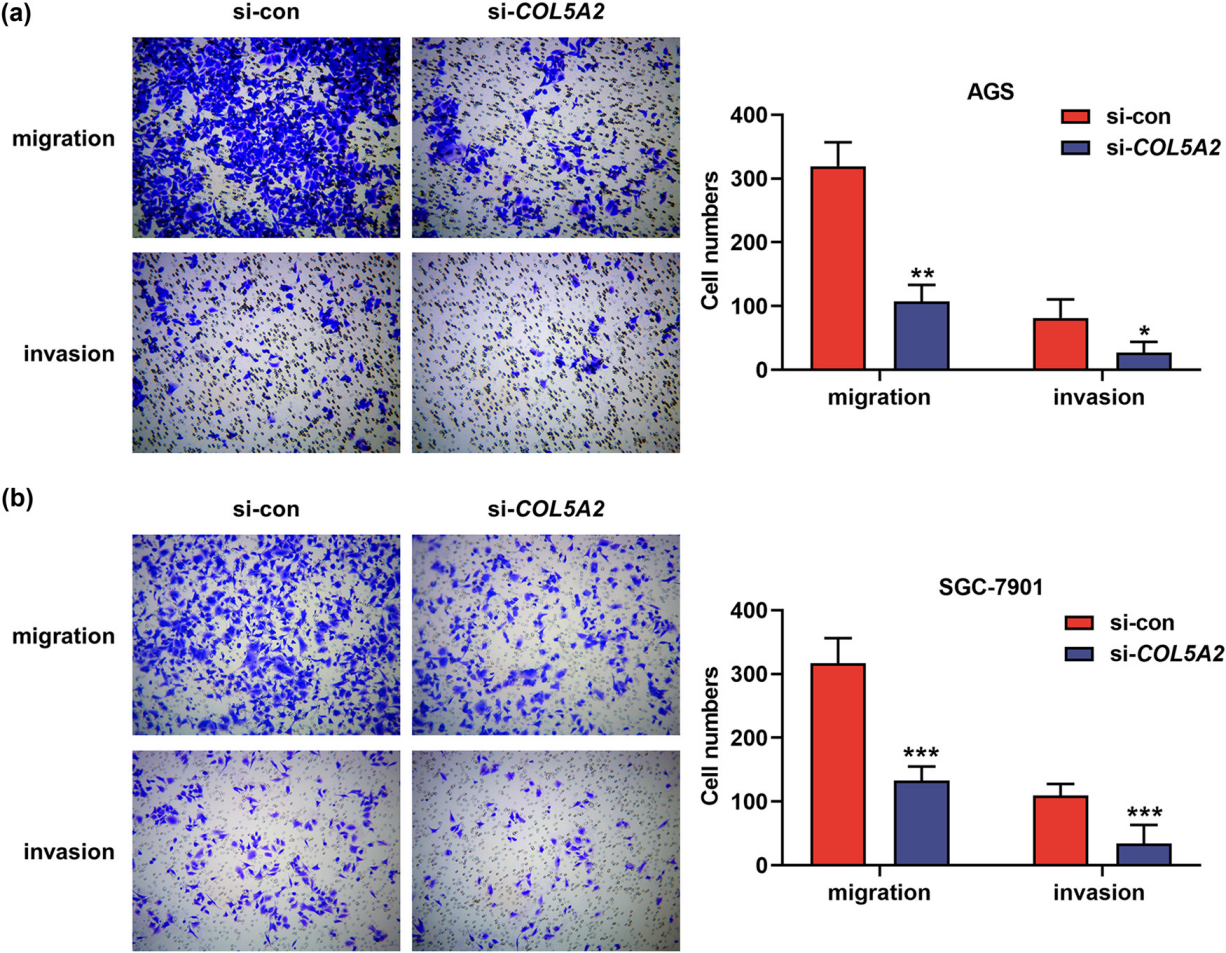
Knockdown of *COL5A2* inhibited the migratory and invasive rates of GC cells. (a and b) Numbers of migratory and invasive cells in si-*COL5A2*- or si-con-transfected AGS (a) and SGC-7901 (b) cells were analyzed by transwell assay. Data from three independent experiments were described as mean ± SD. *P < 0.05, **P < 0.01 and ***P < 0.001 vs si-con group.

Next, cell viability assay and transwell assay were conducted to explore the roles of *COL5A2* in biological behavior of GC cells. As shown in Figure 5a–d, OD values and colony numbers of AGS and SGC-7901 cells were notably decreased in si-*COL5A2* group, as compared with those in si-con group (*P* < 0.01). Besides, AGS and SGC-7901 cells transfected with si-*COL5A2* appeared to decrease in migratory and invasive rates when compared with those transfected with si-con (Figure 6a and b, *P* < 0.01). These results suggested that *COL5A2* positively regulated the proliferation, migration, and invasion of GC cells.

### 3.4 Knockdown of *COL5A2* inhibited biological behaviors of GC cells via regulating EMT-associated genes

**Figure 7:**
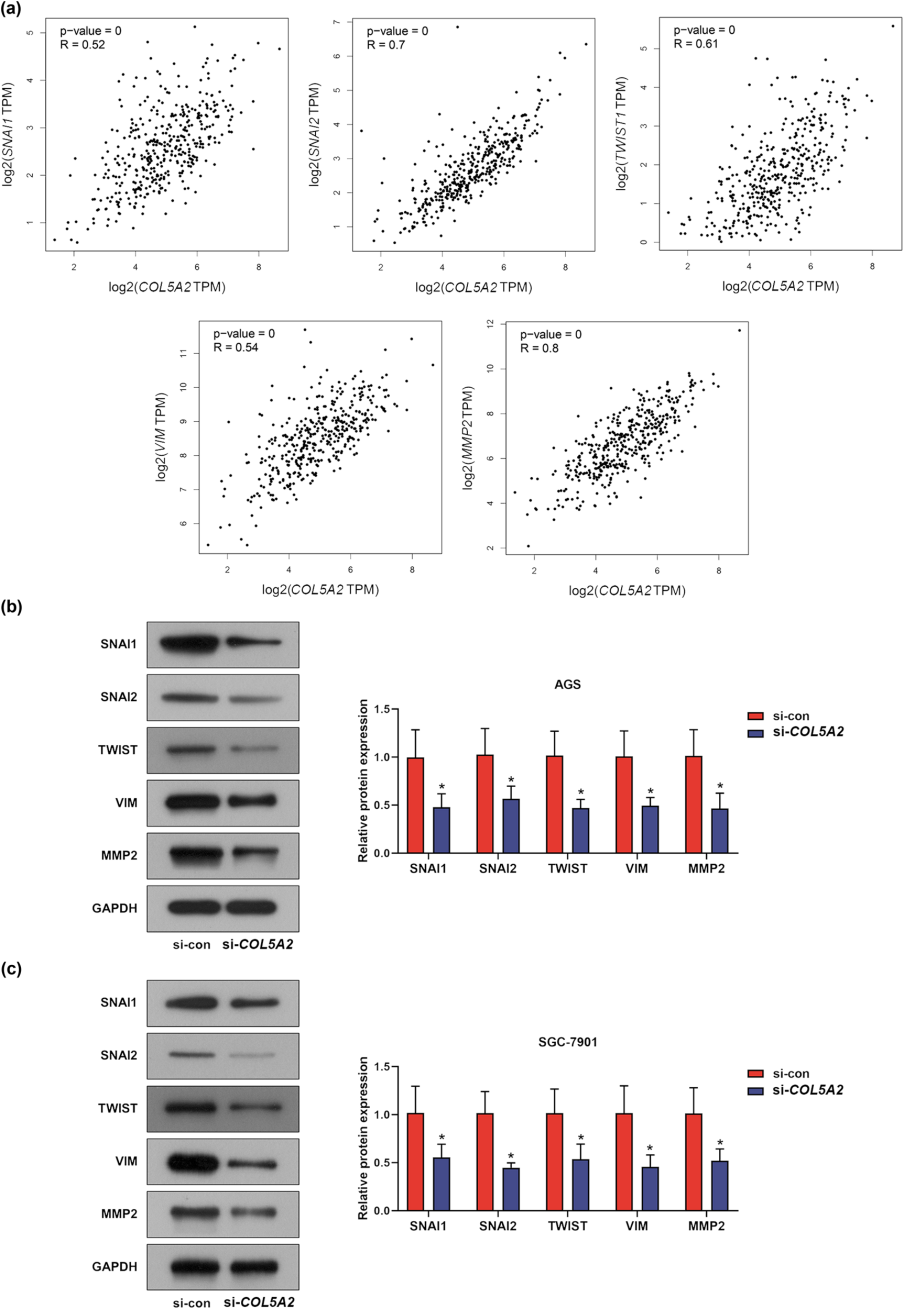
Knockdown of *COL5A2* inhibited biological behaviors of GC cells via regulating EMT-associated genes. (a) Correlation between *COL5A2* expression and EMT-associated genes including *VIM*, *SNAI1*, *SNAI2*, *VIM*, and *MMP2* was analyzed with GEPIA. (b and c) Protein expressions of VIM, SNAI1, SNAI2, VIM, and MMP2 in si-con- or si-*COL5A2*-transfected AGS (b) or SGC-7901 (c) cells were determined by western blot assay. Data from three independent experiments were presented as mean ± SD. *P < 0.05 vs si-con group.

To find out the molecular mechanism on how *COL5A2* regulates the viability, migration, and invasion of GC cells, we evaluated the correlation between *COL5A2* expression and EMT-associated genes including *SNAI1*, *SANI2*, *TWIST1*, *VIM*, and *MMP2* (Figure 7a). First, Spearman’s correlation test revealed that *COL5A2* was positively correlated with the expressions of several mesenchymal markers including *SNAI1* (*P* = 0, *R* = 0.52), *SANI2* (*P* = 0, *R* = 0.7), *TWIST1* (*P* = 0, *R* = 0.61), *VIM* (*P* = 0, *R* = 0.54), and *MMP2* (*P* = 0, *R* = 0.8). Next, western blot was conducted to detect the protein expressions of the above-mentioned mesenchymal markers in *COL5A2*-silenced GC cells. As depicted in Figure 7b and c, the protein expressions of SNAI1, SANI2, TWIST1, VIM, and MMP2 were dramatically decreased in si-*COL5A2* group when compared with those in si-con group (*P* < 0.01). These findings indicated that the knockdown of *COL5A2* could inhibit the protein expressions of EMT-associated markers including SNAI1, SANI2, TWIST1, VIM, and MMP2 to suppress the EMT process of GC.

### 3.5 Overexpression of *COL5A2* promoted the proliferation, migration, invasion, and EMT-associated marker expressions in GC cells

**Figure 8:**
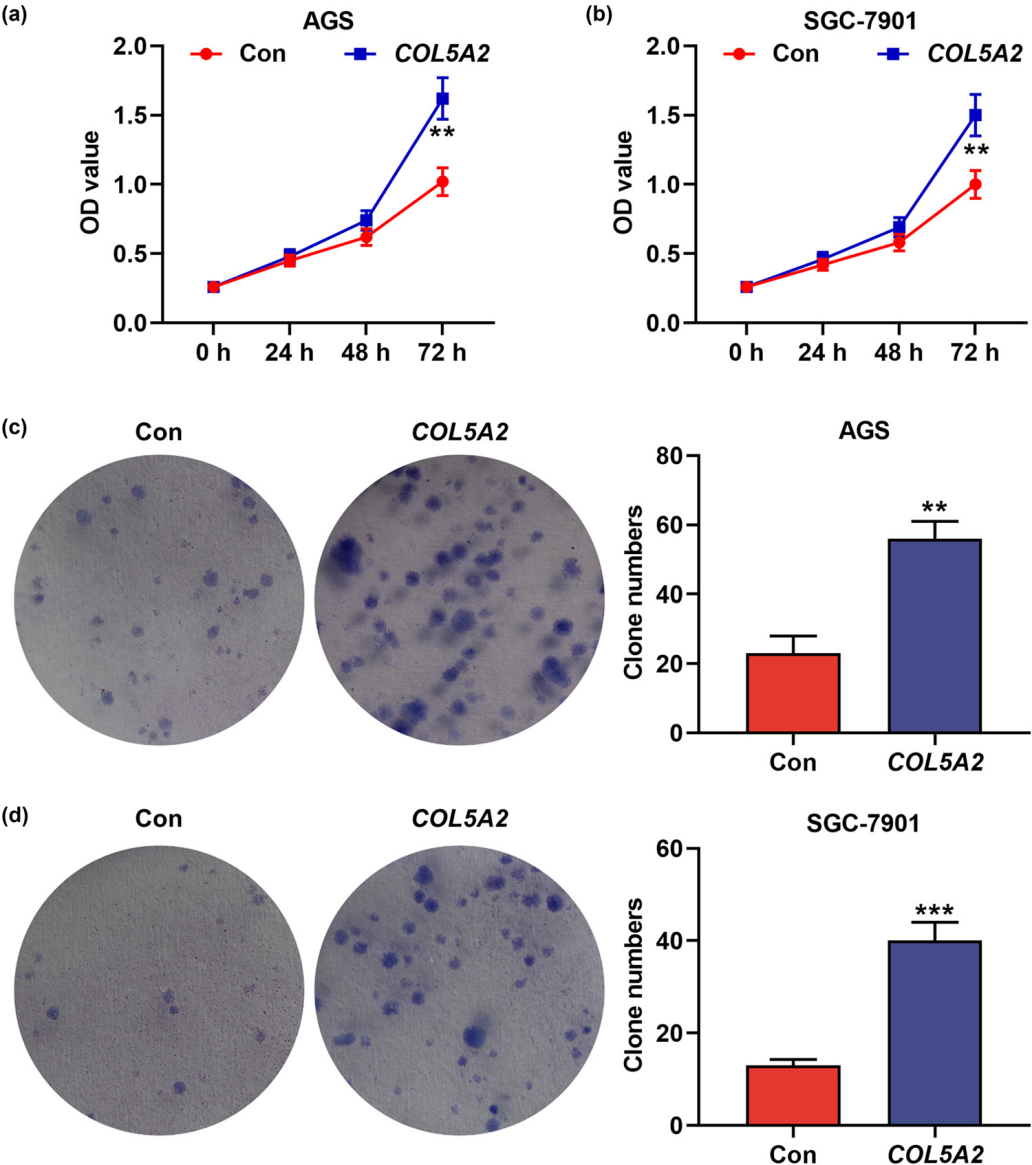
Overexpression of *COL5A2* promoted the proliferation of GC cells. (a and b) After AGS and SGC-7901 cells were transfected with *COL5A2* overexpression plasmid, the viability of AGS (a) and SGC-7901 cells (b) were analyzed by CCK-8 assay. (c and d) After AGS and SGC-7901 cells were transfected with *COL5A2* overexpression plasmid, the colony number of AGS (c) and SGC-7901 cells (d) was analyzed by colony formation assay. **P < 0.01, ***P < 0.001 vs Con group.

**Figure 9:**
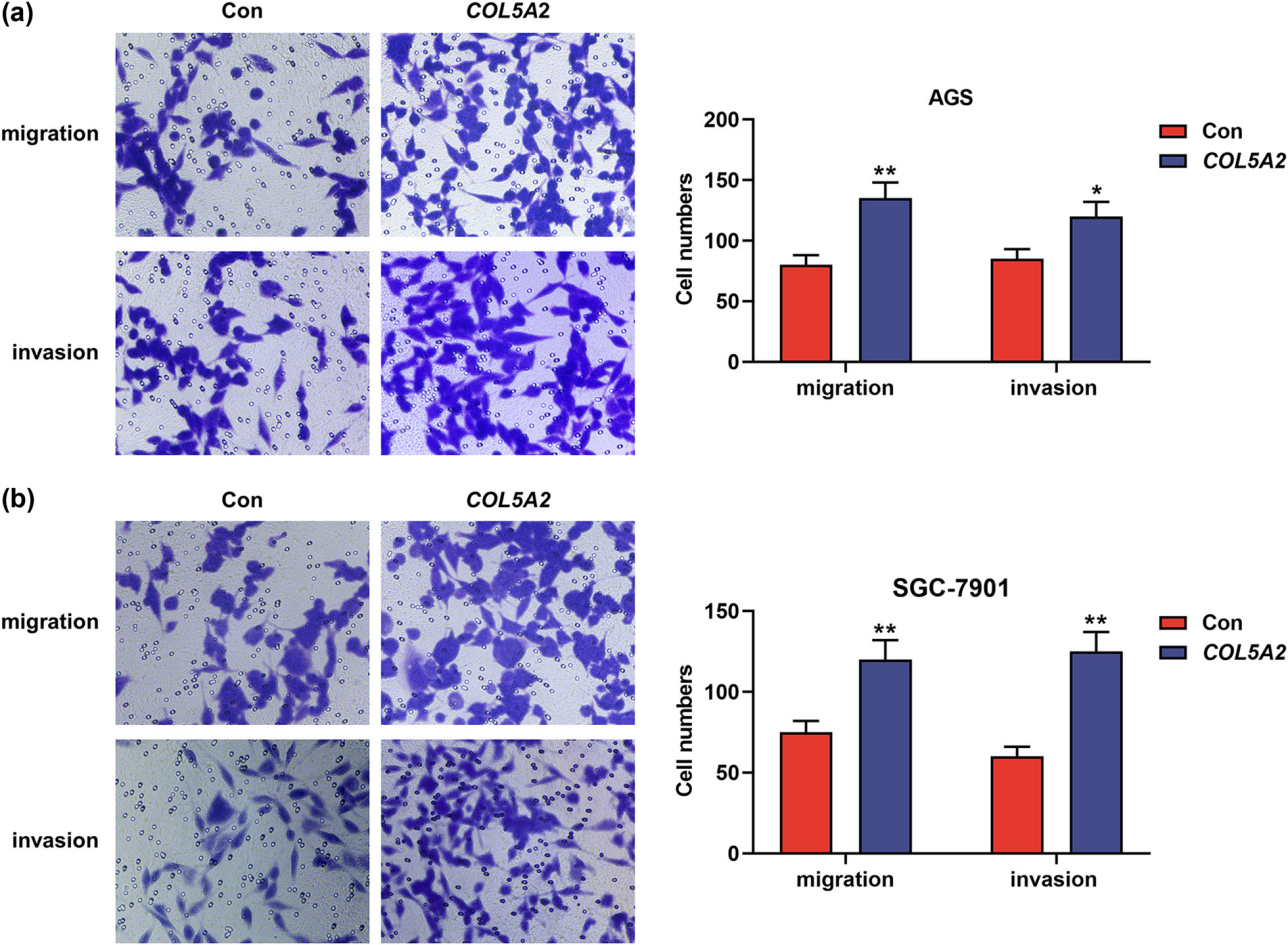
Overexpression of *COL5A2* promoted the migration and invasion of GC cells. (a and b) After AGS and SGC-7901 cells were transfected with *COL5A2* overexpression plasmid, the numbers of migratory and invasive cells in AGS (a) and SGC-7901 cells (b) were analyzed by transwell assay. Data from three independent experiments were exhibited by mean ± SD. *P < 0.05, **P < 0.01 vs Con group.

**Figure 10:**
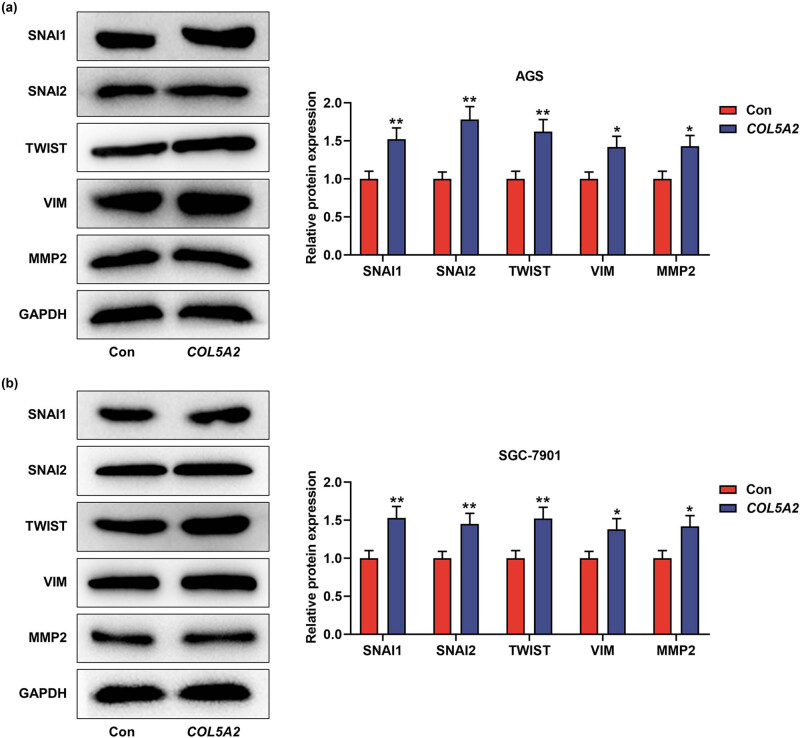
Overexpression of *COL5A2* promoted biological behaviors of GC cells via inducing EMT-associated genes. (a and b) After AGS and SGC-7901 cells were transfected with *COL5A2* overexpression plasmid, the protein expressions of SNAI1, SNAI2, TWIST, VIM, and MMP2 in AGS (a) or SGC-7901 cells (b) were analyzed by western blot assay. Data from three independent experiments were exhibited by mean ± SD. *P < 0.05, **P < 0.01 vs Con group.

In addition, overexpression of *COL5A2* in the GC was also detected. As shown in Figure 8a–d, compared to those in the control group, the OD value and clone number were increased in the *COL5A2* group (*P* < 0.01). Meanwhile, the migration and invasion were increased by overexpressed *COL5A2* (Figure 9a and b, *P* < 0.05). The protein expressions of SNAI1, SANI2, TWIST1, VIM, and MMP2 were higher in COL5A2 group than those in control group (Figure 10a and b, *P* < 0.05). These findings indicated that overexpression of *COL5A2* promoted the proliferation, migration, invasion, and EMT-associated marker expressions in GC.

## 4 Discussion

Multiple bioinformatics analyses have manifested an oncogenic role of *COL5A2* in GC. It has been reported that *COL5A2* was an overexpressed hub gene in GC and associated with the outcome of GC patients [23]. Consistently, our study found that *COL5A2* expression was up-regulated and negatively correlated with the survival percentage of GC. Besides, there was a notable elevation of *COL5A2* expression in stages II, III, and IV compared to that in stage I. Meanwhile, the expression of *COL5A2* was higher in GC in grade III than that in grade I. These results implied that *COL5A2* may play a critical role in the progression of GC.

With the in-depth study of GC, several oncogenes of GC have been found. For example, a high level of legumain was correlated with worse prognosis and peritoneal metastasis in GC patients [24]. C-Maf-inducing protein is an oncogene in human GC cells [25]. Besides, cadherin-6 was highly expressed in GC, and its high expression was correlated with tumor progression and poor prognosis of patients with GC [26]. Additionally, the mRNA expression of *FBXO50* was increased in GC cell lines, and positively correlated with levels of *ITGA5*, *ITGB1*, *MMP*2, *MSN*, *COL5A2*, *GNG11*, and *WNT5A* [27]. In this study, we discovered that *COL5A2* was an oncogenic factor in GC, and silencing of *COL5A2* could repress the proliferation, migration, and invasion of GC cells.

ECM is a key mediator of extracellular microenvironment, which plays an essential role in cancer development by regulating cell transformation, mobility, tumor initiation, and metastasis [28]. Deposited and fibrotic collagens could provide a linearized “tunnel” for cancer cells to migrate and invade [29]. For example, Choi et al. have revealed that weak expression of lysyl oxidase supports the growth and metastasis of breast cancer via regulating the production and remodeling of collagens I and IV [30]. Importantly, *COL5A3* has been reported to enhance proliferative potential in breast cancer cells via binding a membrane proteoglycan GPC-1 [31]. Similarly, in this present study, *COL5A2* potentiated the proliferation, migration, and invasion of GC cells.

As the basic component of ECM, collagens are reported to be involved in the process of EMT [32]. Wei et al. have demonstrated that the accumulation of fibrotic collagens facilitates tumor metastasis by initiating EMT [33]. Besides, collagen type I could augment SNAI1- and LEF-1-mediated EMT in breast cancer [34]. EMT is a cellular process that allows epithelial cells to gain mesenchymal characteristics and entails their mobility via loss of cell adhesion [35]. In this way, EMT facilitates the tumorigenesis, metastasis, and growth of cancers [36]. Recently, accumulating evidence has expounded that there is an intimate correlation among aberrant EMT, cell invasion, and spread of GC [37,38]. EMT is a reversible process involved in two major hallmark proteins, namely adhesion molecules and mesenchymal markers, the former ones contribute to the assembly of epithelial cells and the latter ones are helpful to cancer cell invasion and metastasis [39]. In GC, EMT is induced along with alternation in expressions of various mesenchymal proteins [40]. VIM, a cytoskeletal protein, could promote cell mobility by remodeling cytoskeleton in the process of EMT [41]. SNAI1, SNAI2, and TWIST are three main transcriptional factors regulating EMT [41,42]. Miyoshi et al. have pointed out that SNAI1 could facilitate the invasion of liver cancer cells by elevating the expressions of MMPs [43]. These EMT-associated genes also have been investigated in GC. For example, Zhang et al. have demonstrated that NUB1 blocks EMT process in GC by suppressing the expressions of EMT-related proteins including N-cadherin, VIM, and MMP-2 [44]. AOC1 induces EMT by elevating the expressions of mesenchymal markers including N-cadherin, SNAI1, and SNAI2 and facilitates the progression of GC [45]. Interestingly, similar results were obtained in our study that the protein expressions of VIM, SNAI1, SNAI2, MMP2, and TWIST were decreased by the inhibition of *COL5A2*, suggesting that *COL5A2* could trigger EMT process to regulate the malignant phenotypes of GC. Of course, the effect of *COL5A2* on GC needs to be further elucidated in animal experiments *in vivo*.

In summary, our study reveals that *COL5A2* expression is up-regulated and associated with the dismal outcome of GC patients. Besides, *COL5A2* may be correlated with the higher stages and grades of this disease. *COL5A2* promoted cell growth and mobility of GC. Collectively, *COL5A2* facilitates malignant phenotypes of GC through inducing EMT via promoting the protein expressions of SNAI1, SNAI2, TWIST, VIM, and MMP2, demonstrating the oncogenic role of *COL5A2* in GC and providing a novel clue for the therapeutic application of *COL5A2* in GC.
